# Doppler Examination of the Testicular Artery of Beagle-Breed Dogs from Birth to Puberty

**DOI:** 10.3390/tomography9040112

**Published:** 2023-07-23

**Authors:** Athina P. Venianaki, Mariana S. Barbagianni, George C. Fthenakis, Apostolos D. Galatos, Pagona G. Gouletsou

**Affiliations:** 1 Clinic of Obstetrics and Reproduction, Faculty of Veterinary Science, School of Health Sciences, University of Thessaly, 43100 Karditsa, Greece; 2 Clinic of Surgery, Faculty of Veterinary Science, School of Health Sciences, University of Thessaly, 43100 Karditsa, Greece; 3 Veterinary Faculty, University of Thessaly, 43100 Karditsa, Greece

**Keywords:** blood flow, dog, Doppler, predictor, puberty, semen, testicular artery

## Abstract

The objectives were (a) the study of haemodynamic parameters of blood flow within the testicular artery, (b) the assessment of differences in these parameters at different segments of the artery (i.e., sequentially, as the artery flows through different regions of the testis), and (c) the identification of potential associations with measures of testicular maturation. Eight healthy beagle-breed male dogs were monitored at fortnightly intervals from the 4th to the 40th week of life, by using clinical, seminological, and ultrasonographic (B-mode, pulsed-wave Doppler) examinations. Haemodynamic parameters were assessed at four different segments of the testicular artery: at the distal supra-testicular, the marginal testicular at the cranial pole of the testis, the marginal testicular at the caudal pole of the testis, and the intratesticular. The study period was divided into three time slots (pre-puberty, puberty, and post-puberty) depending on testicular maturation and sperm production. No clinically evident abnormalities were seen in any animal throughout the study. Semen ejaculates were first collected on the 28th week, and spermatozoa were first seen on the 30th week of life. The results of B-mode examination indicated that in all dogs, the echogenicity of the testicular parenchyma was homogeneous. The waveforms of the blood flow in the testicular artery were monophasic with systolic peaks, low diastolic flow, and low vascular resistance. Most cases of significant differences between the three age periods were noted for the comparison of the pre-pubertal to pubertal periods (*n* = 11); among the parameters studied, the blood volume (*n* = 9) showed most instances of significant differences; finally, most cases of significant differences were noted in the distal supra-testicular artery (*n* = 12). Correlations were mainly seen for the end diastolic velocity, the peak systolic velocity and the blood volume (each with two semen evaluation parameters). The distal supra-testicular and the marginal artery at the cranial pole of the testis are recommended as the most appropriate segments of the vessel for performing a Doppler examination in the testicular artery due to the adequate size and the clear spectral waveforms as early as the 12th week of age of the animals.

## 1. Introduction

Ultrasonographic evaluation of the testes is the examination of choice in human and animal clinical practice, as it is a non-invasive, quick, and cost-effective modality that provides information about the position, size, shape, internal architecture, and vascularization of these organs without harming the patient with radiation exposure [1,2,3]. In people, the evaluation of blood flow in the testicular artery by means of the Doppler modality has been found to have high sensitivity and specificity [4] and thus is used for the investigation of epididymitis and testicular torsion [5], disorders often associated with blood flow alternation [6]. In dogs, Doppler examination of the testicular artery can be an additional examination further contributing to diagnosis of subfertility [7].

Doppler examination is useful for imaging the anatomy and function of blood vessels, which can provide information about the presence or absence of blood flow, direction, and flow profile and allow assessment of vascular haemodynamic parameters [8]. In dogs, the method has been used for the assessment of testicular vascularization under physiological or pathological conditions [1,9,10]. It has been also employed in other animal species: rams [11,12], bucks [13,14], stallions [15], jacks [16], bulls [17], and bull camels [18].

Previous studies have shown that the haemodynamic parameters of blood flow within the testicular artery were associated with the testicular volume, spermatogenesis, and semen quality [19,20]. The testicular parenchyma is a highly metabolic tissue, in which any disturbance in nutrient and oxygen supply (through the blood supply) may adversely affect function and morphology [21]. Studies in dogs have shown an association between haemodynamic parameters of blood flow within the testicular artery and semen-related parameters [9,22]; additionally, variations in haemodynamic parameters of blood flow have been reported, as the testicular artery flows through different regions of the testis [23,24]. Further, in rams, it has been proposed to evaluate the artery at the supra-testicular region [25].

In people, characterization of the testicular blood flow profile according to the age of an individual is important [26], as changes in blood flow of the testicular artery have been observed in boys during the peri-puberty period [27]. Thus, the normal range of the testicular arterial blood flow profile for the pre-pubertal, pubertal, and post-pubertal periods has been established [27].

In dogs, only de Souza et al. [28] have reported the possible variations in haemodynamic parameters of blood flow within the testicular artery during the peri-pubertal period; in general, these parameters can be used as indicators of future semen quality [10,22,23]. However, that study did not provide information regarding the evolvement of haemodynamics in the same animals from the pre- to the post-adolescent stage, whilst the animals evaluated were of various breeds, which did not support standardization of results.

In view of the above, a study was instituted and performed in dogs, in the same individuals during pre-puberty, puberty, and post-puberty ages, to provide detailed results of haemodynamic parameters of the testicular artery at different regions of the testes (only for facilitating text flow; thenceforth, these will be termed “segments” of the artery). The objectives were (a) the study of haemodynamic parameters of blood flow within the testicular artery, (b) the assessment of differences in these parameters at different segments of the artery (i.e., sequentially, as the artery flows through different regions of the testis), and (c) the identification of potential associations with measures of testicular maturation.

## 2. Materials and Methods

### 2.1. Animals

Eight healthy, beagle-breed male dogs (from two litters) were used. Initially (for four months), animals were housed with their dam and the other puppies of the litter. Thereafter and until the end of the study, they were penned with the male animals from the same litter. Commercially available dry feed (Royal Canin, Aimargues, Gard, France) was provided twice daily, as appropriate for the age of the animals, and water ad libitum.

Animals were included in the study at the age of two weeks. Before inclusion, a detailed examination confirmed the absence of clinical abnormalities. Moreover, laboratory tests (full haematological and blood and urine biochemical testing) and ultrasonographic assessment were employed to corroborate the health status of animals. The examinations were repeated at monthly intervals throughout the study period. No abnormal findings were seen in any individual throughout the study period.

### 2.2. Ultrasonographic Examination of the Testes

The ultrasonographic examinations of the testes were performed at the same time on each examination day, after the morning feeding, from the 4th to the 40th week of age, every fortnight.

The examination was performed with the animal on lateral recumbency; no sedation was used in any of the imaging procedures. For the examination, the hair of the scrotum was fully clipped. All examinations were performed by the principal investigator (author A.V.) after placing an abundant quantity of coupling gel (Aquasonic^®^ 100; Parker Laboratories, Fairfield, NJ, United States of America) so that no pressure was applied to the scrotum during the examination. Overall, the examination, which covered all regions of both testes of an animal, took 20 to 30 min.

Each testis was imaged separately, beginning with the testicular artery of the right testis with the animal on left lateral recumbency; then, the animal was positioned on right lateral recumbency for examination of the left testis. It was noted that the testicular artery originates directly from the abdominal aorta, and in dogs, it has a complex and tortuous appearance [29]; it was imaged at three different parts of the testis, in accord with the description of Harrison and Weiner [30].

An ultrasound scanner (MyLab^®^ 30; ESAOTE) with a linear transducer with 7.5 to 12.0 MHz imaging frequency was used. Initially, B-mode imaging was performed in order to assess the testicular parenchyma (homogeneity and absence of pathological findings) and to determine the exact anatomical location of the testicular artery; for this, the settings used were 12.0 MHz frequency and 20 to 30 mm scanning depth. The focus was positioned immediately under the distal portion of the *tunica albuginea*. Then, the colour Doppler was switched on, maintaining the pulse-repetition frequency at 1.4 kHz.

**Figure 1:**
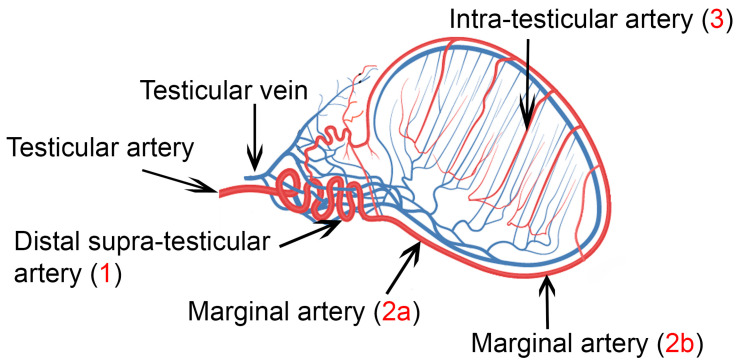
Vasculature of the testis: (**1**) supra-testicular artery, (**2a**) marginal testicular artery at the cranial pole of the testis, (**2b**) marginal testicular artery at the caudal pole of the testis, and (**3**) intratesticular artery.

**Figure 2:**
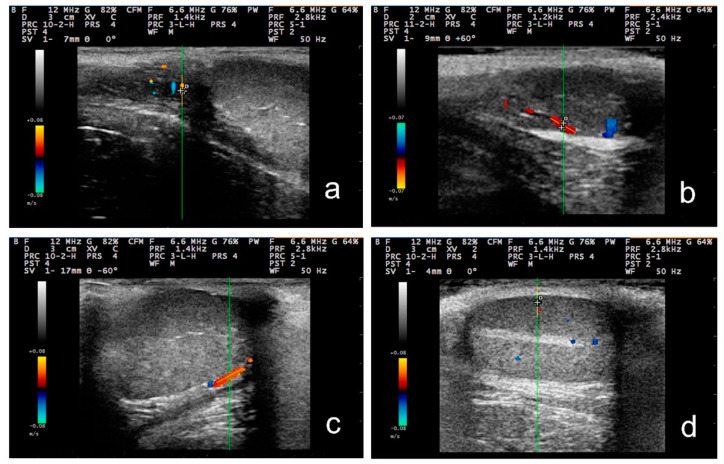
Doppler examination of the right testis of a dog at (**a**) the supra-testicular artery; (**b**) the marginal testicular artery at the cranial pole of the testis; (**c**) the marginal testicular artery at the caudal pole of the testis; (**d**) the intratesticular artery (with 12.0 MHz frequency and 20–30 mm scanning depth).

For the examination, the transducer was positioned initially on the neck of the scrotum, cranially to the testis, to image the distal supra-testicular artery as a looping area. The measurements performed on the distal supra-testicular artery were defined as measurements in “region 1” of the testis (Figure 1 and Figure 2). Then, the transducer, following the artery, was moved to the cranial pole of the testis, and the marginal region of the testicular artery (marginal artery) was imaged in a longitudinal section (defined as “region 2a” of the testis); afterwards, it was moved along the marginal testicular artery to the caudal pole of the testis (defined as “region 2b” of the testis), and further measurements were taken (Figure 1 and Figure 2). Finally, the intratesticular artery in the middle of the testicular parenchyma was imaged in a longitudinal plane (defined as measurements in “region 3” of the testis (Figure 1).

**Figure 3:**
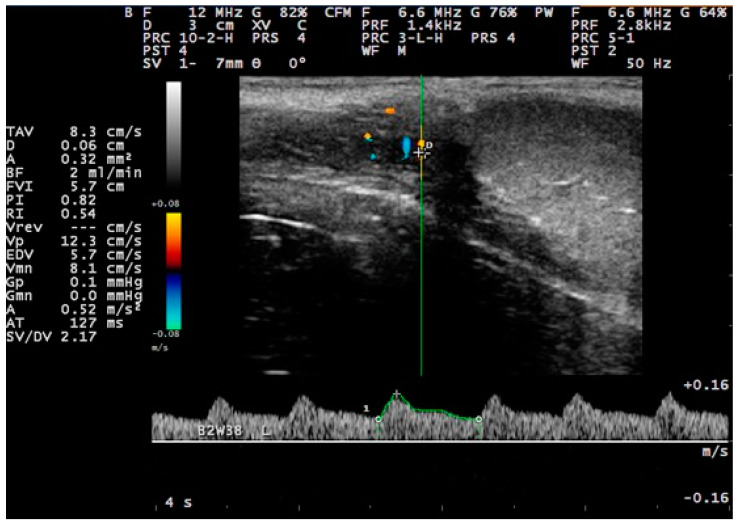
Pulsed-wave Doppler of the distal supra-testicular artery. The diameter of the vessel and the haemodynamic parameters were calculated automatically after manually placing the callipers in the outer walls of the vessel or at the start and end of the cardiac cycle, respectively.

With regard to settings, for the pulsed-wave Doppler examination, 12.0 MHz frequency, 20 to 30 mm examination depth, and pulse-repetition frequency of 1.4 kHz were used. The Doppler filter was set at 100.0 Hz, and the angle of insonation was 40° ± 5° [31]. The colour gain was adjusted in a way to reduce the colour noise (gain 64%), and the Doppler gate was positioned within the lumen of the vessel under examination with no contact with the vessel wall and kept steady at 1 mm. The Doppler angle was between 0° to 60° (Figure 3). At least three continuous and consecutive waves of a cardiac cycle were recorded for the evaluation of haemodynamic parameters.

Seven haemodynamic parameters and the diameter of the vessel were calculated by the equipment software. The haemodynamic parameters calculated were the following: (i) peak systolic velocity (PSV) (indicating the maximum blood speed across the vascular lumen occurring during the systole (m s^−1^)), (ii) end diastolic velocity (EDV) (indicating blood speed at the end of a cardiac cycle (m s^−1^)), (iii) systolic velocity: diastolic velocity ratio (SV/DV) (calculated as average systolic flow: average diastolic flow), (iv) systolic acceleration (A) (indicating blood acceleration across the vascular lumen (m s^−2^)), (v) resistance index (RI) (calculated as ((PSV–EDV)/PSV)), (vi) pulsatility index (PI) (calculated as ((PSV–EDV)/TAMV) (TAMV: time-averaged maximum velocity)), and (vii) blood volume (BF) (indicating the volume of blood that passes through the specific point per unit of time (mL min^−1^)). The diameter of the vessel was calculated after placing the callipers diametrically opposite on the outer wall of the vessel (Figure 3).

### 2.3. Semen Collection and Quality Assessment

The animals were acquainted with the presence of mature female beagle-breed dogs starting at the age of eight weeks. Then, from the age of 10 weeks, they were trained for semen collection by digital manipulation, using a teaser female animal for sexual stimulation. The first semen samples were collected at the age of 28 weeks. Thereafter, semen was collected for evaluation at fortnightly intervals.

A standard seminological evaluation was then performed on the semen samples. The following parameters were assessed: volume of the ejaculate, spermatozoal motility, total number of spermatozoa, viability of spermatozoa, and presence of abnormal spermatozoa, as described in detail by Gouletsou et al. [32].

### 2.4. Data Management and Statistical Analysis

Data were entered into Microsoft Excel (Microsoft Corporation) and analysed using SPSS v. 21 (IBM Analytics). A basic descriptive analysis was performed.

To study changes over time, the period of the experimental study was divided into three age-related periods. The periods were chosen to reflect the different phases of testes maturation and sperm production: the first from birth to the first ejaculation (weeks 4 to 28, “pre-pubertal period”), the second from the first ejaculation to the first recording of spermatozoa in the ejaculate (weeks 30 to 34, “pubertal period”), and the third subsequently to the first recording of spermatozoa in the ejaculate and until the end of the study (weeks 36 to 40, “post-pubertal period”).

Initially, the haemodynamic values of the seven parameters studied and the diameter of the vessel were compared between the left and the right testicular artery for all animals. Haemodynamic values from the left and right testis of an animal were considered together for subsequent analyses.

For each haemodynamic parameter, the results of the three age-related periods were compared between them by using the Kruskal–Wallis test followed by Dunn’s test. Separate analyses were performed for the findings related to each of the four different segments of the testicular artery (“region 1”, “region 2a”, “region 2b”, and “region 3”, as defined above).

The correlation between the semen parameters assessed and the haemodynamic parameters studied was evaluated by Spearman’s correlation analysis. Separate analyses were performed for the findings at each of the four different segments of the testicular artery. For this analysis, only results obtained during the pubertal and the post-pubertal periods were taken into account.

Statistical significance was defined at *p* < 0.05.

## 3. Results

### 3.1. Clinical Examination and Semen Examination

Throughout the study, no clinically evident abnormalities were seen in any animal (e.g., no signs of pain; no scrotal, testicular, or epididymal swelling; no signs related to inflammation or restricted motility of testes within the vaginal cavity). In all dogs, repeated testicular palpation confirmed thin and soft scrotal skin, symmetry between the right and left testis of each animal, physiological consistency of the testes, and size compatibility with the age of the animals.

From all animals, semen ejaculates were first collected on the 28th week of life; spermatozoa were first seen therein on the 30th week of life. The number of spermatozoa progressively increased; from the 36th week of life, total spermatozoa counts in ejaculates exceeded 200 × 10^6^ spermatozoa (Table S1).

### 3.2. B-Mode Examination

The results of B-mode examination indicated that the echogenicity of the testicular parenchyma of all dogs throughout the study was homogeneous. The mediastinum was seen consistently as a thin hyperechoic formation in the centre of the testicular parenchyma, with the testis scanned in a longitudinal plane.

### 3.3. Pulsed-Wave Doppler

Pulsed-wave Doppler measurements were first obtained in the distal supra-testicular artery (region 1 of the testis) from the 12th (3 animals) to 16th (all animals) week, in the marginal artery (region 2 of the testis) from the 18th (2 animals) to 26th (all animals) week, and in the intratesticular artery from the 22nd (1 animal) to 34th (all animals) week. The waveforms of the blood flow in different regions of the testicular artery were monophasic with systolic peaks, low diastolic flow, and low vascular resistance.

There were no significant differences in the haemodynamic values of measurements obtained from the left and right testis of all animals in the study (*p* > 0.65 for all comparisons).

**Figure 4:**
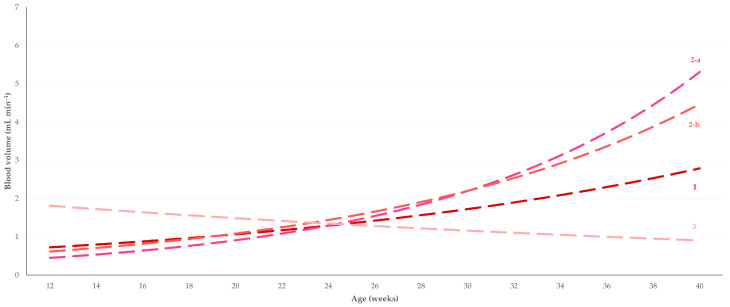
Tendency lines of blood volume (estimated by Doppler ultrasonography) in the testicular artery of young dogs aged 12 to 40 weeks (1, distal supra-testicular artery; 2-a, marginal artery at the cranial pole of the testis; 2-b, marginal artery at the caudal pole of the testis; 3, intratesticular artery).

**Figure 5:**
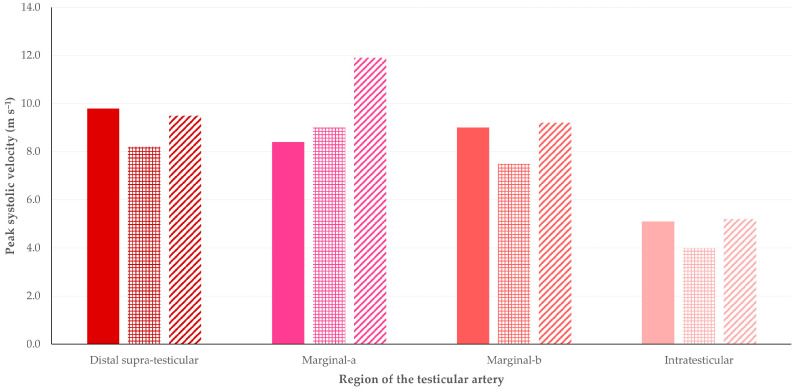
Median values of peak systolic velocity (estimated by Doppler ultrasonography) in different segments of the testicular artery of young dogs throughout three distinct periods of age (solid pattern, 4–28 weeks (pre-pubertal period); square pattern, 30–34 weeks (pubertal period); diagonal pattern, 36–40 weeks (post-pubertal period)).

**Figure 6:**
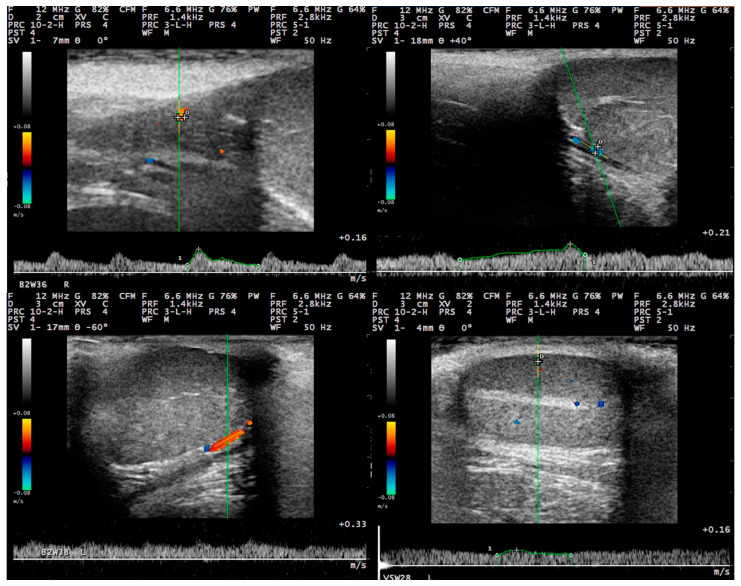
Spectral waveform at the distal supra-testicular artery (**upper left**), the marginal artery at the cranial pole of the testis (**upper right**), the marginal testicular artery at the caudal pole of the testis (**lower left**), and the intratesticular artery (**lower right**) during the post-pubertal period in dogs. Images taken with linear transducer with 12.0 MHz frequency and 20–30 mm scanning depth.

**Figure 7:**
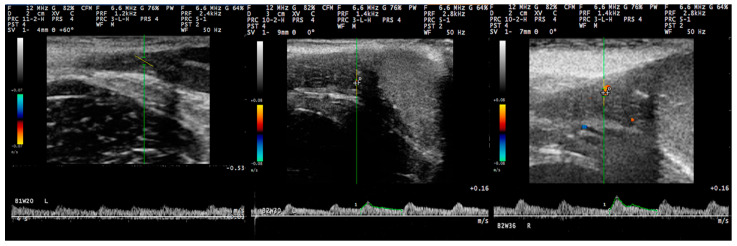
Spectral waveform at the distal supra-testicular artery during the pre-pubertal (**left**), pubertal (**centre**), and post-pubertal period (**right**) in dogs. Images taken with linear transducer with 12.0 MHz frequency and 20–30 mm scanning depth.

Results for the haemodynamic parameters studied, in accord with the study period and the part of the artery imaged on each occasion, are in Table 1. In general, most cases of significant differences between the three age periods were noted for the comparison of the pre-pubertal to the pubertal period (*n* = 11), whilst fewer cases were noted for the comparison of the pubertal to the post-pubertal period (*n* = 9) and the comparison of the pre-pubertal to the post-pubertal period (*n* = 7). Among the parameters studied, the blood volume (*n* = 9) (Figure 4), the vessel diameter (*n* = 6), and the peak systolic velocity (*n* = 4) (Figure 5) showed the most instances of significant differences. Finally, most cases of significant differences were noted in the distal supra-testicular artery (region 1 of the testis) (*n* = 12); in this respect, it is noted that the spectral waveform of blood flow at the distal supra-testicular artery (region 1 of the testis) showed a more prominent systolic peak than in the other regions (Figure 6) in all different time–age-related periods (pre-puberty, puberty, and post-puberty periods) (Figure 7).

### 3.4. Correlations between Semen Evaluation Parameters and Haemodynamic Parameters

During comparison between semen evaluation parameters and haemodynamic parameters, significant correlations were seen only for the findings at the distal supra-testicular segment (region 1 of the testis); correlations were mainly seen for the end diastolic velocity, the peak systolic velocity and the blood volume (each with two semen evaluation parameters) (Table 2). For the other segments of the testicular artery (region 2 and region 3 of the testis), no significant correlations were evident (*p* > 0.085).

## 4. Discussion

### 4.1. Generalities

The ultrasonographic examination of the genital system of male puppies can be particularly useful for the diagnosis of testicular diseases in these animals [33,34].

The pulsed-wave Doppler examination is a useful modality for the assessment of testicular microcirculation status [35]. Indeed, in male dogs, the evaluation of the blood flow within the testicular artery is positively associated with the semen quality and the routine sperm parameters [22,24,36], whilst in bulls, it is associated with the rate of spermatogenesis [37]. Differences in the haemodynamic parameters of blood flow among different segments of the testicular artery have been identified in dogs [23,24]. In rams, the supra-testicular segment has been proposed as the most important region for imaging during the evaluation of the testicular blood flow [25], but in dogs, no relevant reports have been published.

Among the haemodynamic parameters of blood flow evaluated during Doppler examination of the testicular artery, the resistance index (RI) and the pulsatility index (PI) have been studied widely and have been considered as possible indicators of semen quality in dogs [22], rams [38,39], and stallions [15]. Of note, however, is that England et al. [40], in dogs, found no associations between these two parameters and future semen quality.

The resistance index (RI) and the pulsatility index (PI) are ultrasonographic parameters indicating parenchymal perfusion and function of microcirculation in tissues. In the testes, the increase in these indices characterises disruption in the microcirculation and thus a significant reduction in the testicular blood flow. Spermatogenesis is a highly sensitive process in which blood supply is of particular importance, and changes in the blood flow of the area could lead to impairment in sperm production [41]. RI indicates parenchymal perfusion and microvascular function in the testis [42], whilst PI is the measure of the systolic–diastolic differential of the velocity pulse. The RI shows Gaussian distribution and cannot reflect impedance increases, whilst the PI provides more haemodynamic information (includes information on haemodynamic changes during reversed end-diastolic flow) [43]. This is the first study in which all hemodynamic parameters of blood flow in the testicular artery in the same dog breed have been evaluated and compared from birth to maturity. The schedule of the study provided continuous Doppler examinations in the same animals over a long period. Further, the haemodynamic parameters were assessed at different segments of the testicular artery, and thus, recommendations for performing the examination could be discussed.

The spectral waveforms of the testicular artery in all segments imaged were found to be monophasic, with a systolic peak, continual diastolic flow, and low resistive pattern, which is similar to that described in previous studies in dogs [7] and rams [39] and in contrast to findings in stallions, in which species biphasic waveforms of blood flow in the testicular artery within the spermatic cord have been reported [44]. This difference might be the result of variations in the position of the testes in the various species [20], specifically at the supra-testicular artery, as the proximal and medial supra-testicular regions have been found to have higher resistivity patterns compared to the distal one in dogs [45]. The lack of differences in all haemodynamic parameters between the left and right testis, which was taken into account during the data analysis, is in full agreement with the findings of other researchers [7,24], and one may possibly suggest that it is indicative of lack of abnormalities in the testes.

### 4.2. Differences in the Findings between the Regions of the Testis

For most haemodynamic parameters evaluated (RI, PI, systolic acceleration, peak systolic velocity (PSV), and systolic velocity:diastolic velocity ratio (SV/DV)), the higher values were recorded in the distal supra-testicular artery; in contrast, for the end diastolic velocity (EDV), the lower values were recorded there. Similar findings have been reported during the evaluation of mature dogs [24,46] and stallions [44]. An increase in the values of PI and RI is in accord with the decreased perfusion occurring in distal tissues [47]; the low intratesticular capillary pressure present in the testicular parenchyma may also contribute [45].

The significant increase in PSV observed at the marginal artery during the post-puberty period is probably the result of the increased perfusion needs of the epididymis during this period, as the epididymis after puberty has the role of a sperm storage reservoir and sperm maturation [48]. Also, the marginal artery was found to have higher diameter and blood volume compared with other segments of the vessel. This observation may be explained by the straight course of the vessel in this region, a hypothesis that is compatible with similar findings in other animal species [49]. A higher value of PSV in the marginal artery was also reported in studies in mature dogs [24] and jacks [50].

With regard to the intra-testicular artery, where most parameters were found to be at lower levels compared to the other regions, it is noted that in humans, high RI and PI values in the intra-testicular artery have been correlated negatively with sperm quality [51]. In general, increased diastolic flow into the intra-testicular artery has been considered to be the consequence of low vascular resistance of the testis [52,53].

### 4.3. Changes in Accord to Age

The progressive increase in the PSV at the marginal segment of the artery reflects the development of the epididymis and the respective increase in blood volume (BF). This shows a continuous increase with age. This can be attributed to the progressively increasing physiologic vascularization of the testes [54,55].

At the beginning of the study, i.e., shortly after birth, measurements of blood flow in specific segments of the testicular artery cannot be made due to the very small size of the vessels and the very low blood flow. A similar difficulty has been observed in young boys [27]. This might be the result of the small size of the testes and thus the small diameter of the testicular artery, in which the flow cannot be identified [56,57].

### 4.4. Proposed Segments of the Artery and Haemodynamic Parameters for Doppler Evaluation

The two segments of the artery that are proposed for Doppler assessment are the distal supra-testicular artery and the marginal artery at the cranial pole of the testis. The distal supra-testicular artery is the segment most easily accessible for imaging during pre-puberty, when the small size of the testes makes access difficult. The marginal artery is preferred as a straight vessel with an adequate diameter.

Previously, Tarhan et al. [58] suggested assessing the testicular BF at the largest diameter of the testicular artery. It has been reported that PSV and EDV in the supra-testicular artery may not provide very accurate indications as the result of the tortuosity of the vessel [46], but in contrast, Lemos et al. [7] reported that these two haemodynamic parameters were the ones best correlated with sperm concentration in the semen [7].

It has been previously reported that RI and PI can be more reliable indicators than PSV and EDV for the evaluation of blood flow in smaller and more tortuous vessels [59] because the latter parameters can depend on the angle of insonation and the vessel diameter, while values of RI and PI are independent of these factors [59,60]. It has been suggested that RI and PSV could be the most reliable indicators for sperm production since these parameters were highly correlated with “sperm production rate score” [61]. Further, the RI was also proposed by Pozor and McDonnell [44] as the most useful haemodynamic parameter for the functionality of the testes and epididymis, as its values are altered by inflammation [62] and aging [63].

## 5. Conclusions

Natural changes in the haemodynamic parameters of blood flow into the testicular artery occur as puppies grow. These reflect the increased needs of the testicular parenchyma during spermatogenesis and the epididymis for sperm storage and maturation. The changes differ according to the region of the testicular artery, in accord with the regional morphology of the vessel and the intratesticular pressure. The distal supra-testicular artery and the marginal artery at the cranial pole of the testis are recommended as the most appropriate segments of the vessel for performing a Doppler examination in the testicular artery due to the adequate size and the clear spectral waveforms as early as the 12th week of age of the animals.

## Figures and Tables

**Table 1:** Results (median (minimum—maximum) values) for the haemodynamic parameters studied in the testicular artery of 8 young beagle-breed dogs, in accord with the age of animals and the part of the artery imaged on each occasion.

Segment of the Testicular Artery	Age of Animals		
	Weeks 4–28 (Pre-Pubertal Period)	Weeks 30–34 (Pubertal Period)	Weeks 36–40 (Post-Pubertal Period)
Peak Systolic Velocity (m s^−1^)			
Distal supra-testicular (region 1 of the testis)	9.8 (4.9–21.9) ^a^	8.2 (6.2–16.7) ^a,b^	9.5 (6.8–16.4) ^b^
Marginal-a (region 2 of the testis)	8.4 (6.4–14.3) ^a^	9.0 (6.2–12.1) ^b^	11.9 (6.2–16.1) ^a,b^
Marginal-b (region 2 of the testis)	9.0 (4.4–15.9)	7.5 (7.0–14.4)	9.2 (7.0–18.0)
Intratesticular (region 3 of the testis)	5.1 (3.2–8.5)	4.0 (2.9–8.1)	5.2 (5.0–5.4)
End Diastolic Velocity (m s^−1^)			
Distal supra-testicular (region 1 of the testis)	4.2 (1.9–8.1) ^a^	3.3 (2.6–6.3) ^a^	5.0 (2.6–7.7)
Marginal-a (region 2 of the testis)	5.0 (2.9–8.2)	5.2 (3.1–6.8)	5.8 (3.1–10.0)
Marginal-b (region 2 of the testis)	4.9 (2.6–8.6)	4.6 (3.5–8.1)	5.6 (3.5–7.1)
Intratesticular (region 3 of the testis)	5.8 (3.6–9.3)	4.2 (3.1–8.9)	5.3 (5.3–5.3)
Systolic: Diastolic Velocity Ratio			
Distal supra-testicular (region 1 of the testis)	2.3 (1.6–4.3) ^a^	2.8 (1.7–4.1) ^a^	2.4 (1.7–3.7)
Marginal-a (region 2 of the testis)	1.9 (1.4–2.3)	1.9 (1.5–2.5)	1.9 (1.5–2.4)
Marginal-b (region 2 of the testis)	1.9 (1.3–3.0)	1.8 (1.6–2.0) ^a^	2.1 (1.6–2.5) ^a^
Intratesticular (region 3 of the testis)	1.3 (1.1–1.9)	1.4 (1.1–1.8)	1.1 (1.1–1.1)
Systolic Acceleration (m s^−2^)			
Distal supra-testicular (region 1 of the testis)	0.45 (0.20–3.39) ^a^	0.41 (0.32–0.89) ^a^	0.41 (0.31–1.06)
Marginal-a (region 2 of the testis)	0.35 (0.19–0.50)	0.35 (0.22–0.40)	0.42 (0.22–0.64)
Marginal-b (region 2 of the testis)	0.30 (0.06–0.50)	0.29 (0.20–0.65)	0.35 (0.20–0.71)
Intratesticular (region 3 of the testis)	0.18 (0.04–0.25)	0.20 (0.01–0.25)	0.05 (0.05–0.05)
Resistance Index			
Distal supra-testicular (region 1 of the testis)	0.68 (0.35–1.45) ^a^	0.71 (0.41–1.70) ^a^	0.62 (0.41–1.37)
Marginal-a (region 2 of the testis)	0.45 (0.24–0.61)	0.42 (0.32–0.60)	0.50 (0.32–0.58)
Marginal-b (region 2 of the testis)	0.44 (0.20–0.67)	0.42 (0.38–0.50) ^a^	0.47 (0.38–0.61) ^a^
Intratesticular (region 3 of the testis)	0.10 (0.09–0.37)	0.09 (0.06–0.22)	0.08 (0.06–0.10)
Pulsatility Index			
Distal supra-testicular (region 1 of the testis)	0.84 (0.44–1.70) ^a^	1.10 (0.52–1.70) ^a^	0.89 (0.52–1.37)
Marginal-a (region 2 of the testis)	0.58 (0.33–0.89)	0.54 (0.40–1.03)	0.71 (0.40–0.88)
Marginal-b (region 2 of the testis)	0.57 (0.24–1.14)	0.49 (0.58–0.74) ^a^	0.65 (0.49–1.01) ^a^
Intratesticular (region 3 of the testis)	0.11 (0.09–0.46)	0.09 (0.07–0.25)	0.08 (0.07–0.09)
Blood Volume (mL min^−1^)			
Distal supra-testicular (region 1 of the testis)	1 (1–5) ^a,b^	2 (1–5) ^a,c^	3 (1–9) ^b,c^
Marginal-a (region 2 of the testis)	1 (1–3) ^a,b^	2.5 (1–9) ^a,c^	4.5 (2–9) ^b,c^
Marginal-b (region 2 of the testis)	1 (1–5) ^a,b^	1 (1–5) ^a,c^	5 (1–7) ^b,c^
Intratesticular (region 3 of the testis)	1 (1–1)	1 (1–2)	1 (1–1)
Diameter (cm)			
Distal supra-testicular (region 1 of the testis)	0.02 (0.01–0.30) ^a,b^	0.08 (0.03–0.80) ^a^	0.08 (0.05–0.14) ^b^
Marginal-a (region 2 of the testis)	0.06 (0.01–0.10) ^a^	0.09 (0.04–0.70)	0.12 (0.08–0.15) ^a^
Marginal-b (region 2 of the testis)	0.01 (0.01–0.14) ^a,b^	0.07 (0.01–0.12) ^a,c^	0.11 (0.03–0.11) ^b,c^
Intratesticular (region 3 of the testis)	0.01 (0.01–0.09)	0.02 (0.01–0.09)	0.04 (0.01–0.06)

^a,b,c^ Superscripts within the same row indicate differences between the respective age periods.

**Table 2:** Spearman’s rank correlation coefficient (significance of correlation (*p*-values)) found between the semen parameters assessed and the haemodynamic parameters studied at the distal supra-testicular artery in eight beagle-breed dogs.

Haemodynamic Parameters	Semen Evaluation Parameters		
	Motility	Total No. of Spermatozoa in Ejaculate	Sperm Viability
Peak Systolic Velocity	0.415 (0.044)	0.436 (0.016)	ns
End Diastolic Velocity	0.490 (0.014)	0.520 (0.003)	ns
Systolic: Diastolic Velocity Ratio	ns ^1^	ns	ns
Systolic Acceleration	ns	ns	ns
Resistance Index	ns	−0.335 (0.047)	ns
Pulsatility Index	ns	ns	ns
Blood Volume	0.519 (0.010)	0.493 (0.007)	ns
Diameter	ns	ns	ns

^1^ ns, not significant (*p* ≥ 0.05).

## Data Availability

Most data presented in this study are in the text or in the Supplementary Materials. The remaining data are available on request from the corresponding author. The data are not publicly available, as they form part of the Ph.D. thesis of the first author, which has not yet been examined, approved, and uploaded in the official depository of Ph.D. theses from Greek universities.

## Supplementary Materials

The following supporting information can be downloaded at: https://www.mdpi.com/article/10.3390/tomography9040112/s1, Table S1: Results of semen evaluation parameters in 8 young beagle-breed dogs, in accord with the age of the animals.

## References

[1] De Souza Μ.Β., Silva L.D., Moxon R., Russo M., England G.C.W. (2017). Ultrasonography of the prostate gland and testes in dogs. In Pract..

[2] Spaziani M., Lecis C., Tarantino C., Sbardella E., Pozza C., Gianfrilli D. (2021). The role of scrotal ultrasonography from infancy to puberty. Andrology.

[3] Orlandi R., Vallesi E., Boiti C., Polisca A., Bargellini P., Troisi A. (2022). Characterization of testicular tumor lesions in dogs by different ultrasound techniques. Animals.

[4] Dogra V.S., Gottlieb R.H., Oka M., Rubens D.J. (2003). Sonography of the scrotum. Radiology.

[5] Lerner R.M., Mevorach R.A., Hulbert W.C., Rabinowitz R. (1990). Color Doppler US in the evaluation of acute scrotal disease. Radiology.

[6] Akand M., Koplay M., Islamoglu N., Altintas E., Kilic O., Gul M., Kulaksizoglu H., Sivri M., Goktas S. (2017). Color Doppler ultrasound characteristics after subinguinal microscopic varicocelectomy. Med. Ultrason..

[7] Lemos H., Dorado J., Hidalgo M., Gaivão I., Martins-Bessa A. (2020). Assessment of dog testis perfusion by colour and pulsed-doppler ultrasonography and correlation with sperm oxidative DNA damage. Top. Companion Anim. Med..

[8] Mattoon J.S., Nyland T.G., Mattoon J.S., Nyland T.G. (2015). Chapter 17—Prostate and Testes. Small Animal Diagnostic Ultrasound.

[9] de Souza M.B., England G.C., Mota Filho A.C., Ackermann C.L., Sousa C.V., de Carvalho G.G., Silva H.V., Pinto J.N., Linhares J.C., Oba E. (2015). Semen quality, testicular B-mode and Doppler ultrasound, and serum testosterone concentrations in dogs with established infertility. Theriogenology.

[10] Trautwein L.G.C., Souza A.K., Cardoso G.S., da Costa Flaiban K.K.M., de Oliveira Dearo A.C., Martins M.I.M. (2020). Correlation of testicular artery Doppler velocimetry with kinetics and morphologic characteristics of epididymal sperm in dogs. Reprod. Dom. Anim..

[11] Hedia M.G., El-Belely M.S., Ismail S.T., Abo El-Maaty A.M. (2019). Monthly changes in testicular blood flow dynamics and their association with testicular volume, plasma steroid hormones profile and semen characteristics in rams. Theriogenology.

[12] Hassan M.A.A., Sayed R.K.A., Abdelsabour-Khalaf M., Abd-Elhafez E.A., Anel-Lopez L., Riesco M.F., Ortega-Ferrusola C., Montes-Garrido R., Neila-Montero M., Anel L. (2022). Morphological and ultrasonographic characterization of the three zones of supratesticular region of testicular artery in Assaf rams. Sci. Rep..

[13] Samir H., Nyametease P., Elbadawy M., Nagaoka K., Sasaki K., Watanabe G. (2020). Administration of melatonin improves testicular blood flow, circulating hormones, and semen quality in Shiba goats. Theriogenology.

[14] Abdelkhalek K.G., Badawy A.B.A., Abdelnaby E.A., Fathi M. (2022). Relationship between main testicular hemodynamics and computerassisted analysis in prepubertal age for breeding selection in Baladi bucks. J. Adv. Vet. Res..

[15] Ortiz-Rodríguez J.M., Anel-Lopez L., Martín-Muñoz P., Álvarez M., Gaitskell-Phillips G., Anel L., Rodríguez-Medina P., Peña F.J., Ferrusola O. (2017). Pulse Doppler ultrasound as a tool for the diagnosis of chronic testicular dysfunction in stallions. PLoS ONE.

[16] Abdelnaby E.A., Emam I.A., Fadl A.M. (2021). Assessment of the accuracy of testicular dysfunction detection in male donkey (*Equus asinus*) with the aid of colour-spectral Doppler in relation to plasma testosterone and serum nitric oxide levels. Reprod. Dom. Anim..

[17] Gloria A., Carluccio A., Wegher L., Robbe D., Valorz C., Contri A. (2018). Pulse wave Doppler ultrasound of testicular arteries and their relationship with semen characteristics in healthy bulls. J. Anim. Sci. Biotechnol..

[18] Kutzler M., Tyson R., Grimes M., Timm K. (2011). Determination of testicular blood flow in camelids using vascular casting and color pulsed-wave Doppler ultrasonography. Vet. Med. Int..

[19] Pozor M.A. (2007). Evaluation of testicular vasculature in stallions. Clin. Tech. Equine Pract..

[20] Velasco A., Ruiz S. (2020). New approaches to assess fertility in domestic animals: Relationship between arterial blood flow to the testicles and seminal quality. Animals.

[21] Colli L.G., Belardin L.B., Echem C., Akamine E.H., Antoniassi M.P., Andretta R.R., Mathias L.S., Rodrigues S.F.P., Bertolla R.P., de Carvalho M.H.C. (2019). Systemic arterial hypertension leads to decreased semen quality and alterations in the testicular microcirculation in rats. Sci. Rep..

[22] Zelli R., Troisi A., Elad Ngonput A., Cardinali L., Polisca A. (2013). Evaluation of testicular artery blood flow by Doppler ultrasonography as a predictor of spermatogenesis in the dog. Res. Vet. Sci..

[23] Carrillo J.D., Soler M., Lucas X., Agut A. (2012). Colour and pulsed Doppler ultrasonographic study of the canine testis. Reprod. Dom. Anim..

[24] de Souza M.B., da Cunha Barbosa C., Pereira B.S., Monteiro C.L., Pinto J.N., Linhares J.C., da Silva L.D. (2014). Doppler velocimetric parameters of the testicular artery in healthy dogs. Res. Vet. Sci..

[25] Ntemka A., Kiossis E., Boscos C., Theodoridis A., Kourousekos G., Tsakmakidis I. (2018). Effects of testicular hemodynamic and echogenicity changes on ram semen characteristics. Reprod. Dom. Anim..

[26] Wood M.M., Romine L.E., Lee Y.K., Richman K.M., O’Boyle M.K., Paz D.A., Chu P.K., Pretorius D.H.D.A. (2010). Spectral Doppler signatures waveforms in ultrasonography. Ultrasound Q..

[27] Paltiel H.J., Rupich R.C., Babcock D.S. (1994). Maturational changes in arterial impedance of the normal testis in boys: Doppler sonographic study. Am. J. Roentgenol..

[28] de Souza M.B., Barbosa C.C., England G.C., Mota Filho A.C., Sousa C.V., de Carvalho G.G., Silva H.V., Pinto J.N., Linhares J.C., Silva L.D. (2015). Regional differences of testicular artery blood flow in post pubertal and pre-pubertal dogs. BMC Vet. Res..

[29] Baez A.D., Cabrera W.R., Llano E.G. (2007). Origen de las arterias testiculares en caninos. Rev. Veter..

[30] Harrison R.G., Weiner J.S. (1949). Vascular patterns of the mammalian testis and their functional significance. J. Exp. Biol..

[31] Strina A., Corda A., Nieddu S., Solinas G., Lilliu M., Zedda M.T., Pau S., Ledda S. (2016). Annual variations in resistive index (RI) of testicular artery, volume measurements and testosterone levels in bucks. Comp. Clin. Pathol..

[32] Gouletsou P.G., Galatos A.D., Leontides L.S., Sideri A.I. (2011). Impact of fine- or large-needle aspiration on canine testes: Clinical, in vivo ultrasonographic and seminological assessment. Reprod. Dom. Anim..

[33] Cicirelli V., Aiudi G.G., Carbonara S., Caira M., Lacalandra G.M. (2021). Case of anorchia in a mixed-breed dog. Top. Companion Anim. Med..

[34] Cicirelli V., Burgio M., Mrenoshki D., Cseh S., Aiudi G., Lacalandra G.M. (2023). Update on canine anorchia: A review. Vet. Med. Sci..

[35] Semiz I., Tokgöz O., Tokgoz H., Voyvoda N., Serifoglu I., Erdem Z. (2014). The investigation of correlation between semen analysis parameters and intraparenchymal testicular spectral Doppler indices in patients with clinical varicocele. Ultrasound Q..

[36] Moxon R., Bright L., Pritchard B., Bowen I.M., de Souza M.B., da Silva L.D., England G.C. (2015). Digital image analysis of testicular and prostatic ultrasonographic echogencity and heterogeneity in dogs and the relation to semen quality. Anim. Reprod. Sci..

[37] Kay G.W., Grobbelaar J.A., Hattingh J. (1992). Effect of surgical restric-tion of growth of the testicular artery on testis size and histology in bulls. J. Reprod. Fert..

[38] Batissaco L., Celeghini E.C.C., Pinaffi F.L.V., Oliveira B.M.M., de Andrade A.F.C., de Recalde E.C.S., Fernandes C.B. (2013). Correlations between testicular hemodynamic and sperm characteristics in rams. Vet. Res. Anim. Sci..

[39] Elbaz H., Elweza A.E., Sharshar A.M. (2019). Testicular color doppler ultrasonography in Barki rams. Alex. J. Vet. Sci..

[40] England G., Bright L., Pritchard B., Bowen I.M., de Souza M.B., Silva L., Moxon R. (2017). Canine reproductive ultrasound examination for predicting future sperm quality. Reprod. Dom. Anim..

[41] Srivastava S., Panchal S., Nagori C., Thaker M. (2021). Doppler parameters of intratesticular vessels in male factor sub fertility. Andrology.

[42] Aguilar-García J., Cano-González H., Martínez-Jiménez M., de la Rosa-Zapata F., Sánchez-Aguilar M. (2018). Unilateral Lichtenstein tension-free mesh hernia repair and testicular perfusion: A prospective control study. Hernia.

[43] Maulik D., Maulik D. (2005). Spectral Doppler sonography. Waveform analysis and hemodynamic interpretation. Doppler Ultrasound in Obstetrics and Gynecology.

[44] Pozor M.A., McDonnell S.M. (2004). Color Doppler ultrasound evaluation of testicular blood flow in stallions. Theriogenology.

[45] Trautwein L.G.C., Souza A.K., Martins M.I.M. (2019). Can testicular artery Doppler velocimetry values change according to the measured region in dogs?. Reprod. Dom. Anim..

[46] Gumbsch P., Holzmann A., Gabler C. (2002). Colour-coded duplex sonography of the testes in dogs. Vet. Rec..

[47] Samir H., ElSayed M.I., Radwan F., Hedia M., Hendawy H., Hendawy A.O., Elbadawy M., Watanabe G. (2023). An updated insight on testicular hemodynamics: Environmental, physiological, and technical perspectives in farm and companion animals. Vet. Res. Commun..

[48] Ahmadi B., Lau C.P., Giffin J., Santos N., Hahnel A., Raeside J., Christie H., Bartlewski P. (2012). Suitability of epididymal and testicular ultrasonography and computerized image analysis for assessment of current and future semen quality in the ram. Exp. Biol. Med..

[49] Elayat M.A., Khalil K.M., Farag F.M., Rizk H.M. (2014). Gross anatomical studies on the pattern and density of the tunica vasculosa testis in some farm animals (buffalo, ram, camel donkey and rabbit). Benha Med. J..

[50] Gacem S., Papas M., Catalan J., Miró J. (2020). Examination of jackass (Equus asinus) accessory sex glands by B-mode ultrasound and of testicular artery blood flow by colour pulsed-wave Doppler ultrasound: Correlations with semen production. Reprod. Dom. Anim..

[51] Pinggera G.M., Mitterberger M., Bartsch G., Strasser H., Gradl J. (2008). Assessment of the intratesticular resistive index by colour Doppler ultrasonography measurements as a predictor of spermatogenesis. BJU Int..

[52] Middleton W.D., Thorne D.A., Melson G.L. (1989). Color Doppler ultrasound of the normal testis. Am. J. Roentgenol..

[53] Ortega-Ferrusola C., Gracia-Calvo L.A., Ezquerra J., Pena F.J. (2014). Use of colour and spectral Doppler ultrasonography in stallion andrology. Reprod. Dom. Anim..

[54] Saunders H.M., Burns P.N., Needleman L., Liu J.B., Boston R., Wortman J.A., Chan L. (1998). Hemodynamic factors affecting uterine artery Doppler waveform pulsatility in sheep. J. Ultrasound Med..

[55] Dudea S.M., Ciurea A., Chiorean A., Botar-Jid C. (2010). Doppler applications in testicular and scrotal disease. Med. Ultrason..

[56] Luker G.D., Siegel M.J. (1996). Scrotal US in pediatric patients: Comparison of power and standard color Doppler US. Radiology.

[57] Bader T.R., Kammerhuber F., Herneth A.M. (1997). Testicular blood flow in boys as assessed at color Doppler and power Doppler sonography. Radiology.

[58] Tarhan S., Ucer O., Sahin M.O., Gumus B. (2010). Long-term effect of microsurgical inguinal varicocelectomy on testicular blood flow. J. Androl..

[59] Marchesini A.C., Magrio F.A., Berezowski A.T., Neto O.B., Nogueira A.A., Candido dos Reis F.J. (2008). A critical analysis of Doppler velocimetry in the differential diagnosis of malignant and benign ovarian masses. J. Wom. Health.

[60] Sladkevicius P., Valentin L. (1995). Reproducibility of doppler measurements of blood flow velocity in the uterine and ovarian arteries in premenopausal women. Ultrasound Med. Biol..

[61] Biagiotti G., Cavallini G., Modenini F., Vitali G., Gianaroli L. (2002). Spermatogenesis and spectral echo-colour Doppler traces from the main testicular artery. BJU Int..

[62] Jee W.H., Choe B.Y., Byun J.Y., Shinn K.S., Hwang T.K. (1997). Resistive index of the intrascrotal artery in scrotal inflammatory disease. Acta Radiol..

[63] Wielgoś M., Bablok L., Fracki S., Marianowski L. (1998). Doppler flowmetry measurements in testicular artery of aging men. Ginekol. Pol..

