# Supplementary material for: Doppler Examination of the Testicular Artery of Beagle-Breed Dogs from Birth to Puberty
Source: Tomography. 2023 Jul 23;9(4):1408–22. doi: 10.3390/tomography9040112 (PMC10366859; doi:10.3390/tomography9040112)
Supplement: Supplementary file 1 [file tomography-09-00112-s001.zip › tomography-2502927-SI.pdf]

Doppler Examination of the Testicular Artery of Beagle-Breed Dogs from Birth to Puberty

Athina P. Venianaki, Mariana S. Barbagianni, George C. Fthenakis, Apostolos D. Galatos and Pagona G. Gouletsou

Table S1. Results (median (minimum – maximum) values) of semen evaluation parameters in 8 young Beagle-breed dogs, in accord with the age of the animals.

	**Age of animals**	
**Semen evaluation parameter**	Weeks 30-34 (pubertal period)	Weeks 36-40 (post-pubertal period)
Volume (mL)	0.37 (0.06 – 1.37)	1.24 (0.35 – 3.54)
Motility (%)	70 (50 - 80)	90 (80 – 100)
Spermatozoa concentration (mL^{-1})	7.5×10^6 (1.0×10^6 – 188.5×10^6)	477.0×10^6 (204.0×10^6 – 1155.0×10^6)
Total spermatozoa in ejaculate	3.7×10^6 (0.3×10^6 – 120.6×10^6)	636.1×10^6 (229×10^6 – 1313.3×10^6)
Sperm viability (%)	97.5 (80 – 100)	100 (95 – 100)
